# Effect of intra-arterial nimodipine on iatrogenic vasospasms during endovascular stroke treatment – angiographic resolution and infarct growth in follow-up imaging

**DOI:** 10.1186/s12883-022-03045-x

**Published:** 2023-01-05

**Authors:** Jessica Jesser, Arne Potreck, Dominik Vollherbst, Fatih Seker, Min Chen, Silvia Schönenberger, Thuy D. Do, Martin Bendszus, Markus A. Möhlenbruch, Charlotte S. Weyland

**Affiliations:** 1 Department of Neuroradiology, Heidelberg University Hospital, Im Neuenheimer Feld 400, 69120 Heidelberg, Germany; 2 Department of Neurology, Heidelberg University Hospital, Im Neuenheimer Feld 400, 69120 Heidelberg, Germany; 3 Department of Diagnostic and Interventional Radiology, Heidelberg University Hospital, Im Neuenheimer Feld 410, 69120 Heidelberg, Germany

**Keywords:** Endovascular stroke treatment, Thrombectomy, Vasospasm, Nimodipine, Intracranial hemorrhage, ASPECTS

## Abstract

**Purpose:**

The treatment of vasospasms during endovascular stroke treatment (EST) with intra-arterial nimodipine (NM) is routinely performed. However, the efficacy of resolving iatrogenic vasospasms during the angiographic intervention and the infarct development in follow-up imaging after EST has not been studied yet.

**Methods:**

Retrospective single-center analysis of patients receiving EST for anterior circulation vessel occlusion between 01/2015 and 12/2021. The primary endpoint was ASPECTS in follow-up imaging. Secondary endpoints were the clinical outcome (combined endpoint NIHSS 24 h after EST and difference between modified Rankin Scale (mRS) before stroke and at discharge (delta mRS)) and intracranial hemorrhage (ICH) in follow-up imaging. Patients with vasospasms receiving NM (NM+) or not (NM-) were compared in univariate analysis.

**Results:**

Vasospasms occurred in 79/1283 patients (6.2%), who consecutively received intra-arterial NM during EST. The targeted vasospasm angiographically resolved in 84% (66/79) under NM therapy. ASPECTS was lower in follow-up imaging after vasospasms and NM-treatment (NM – 7 (6–9), NM + 6 (4.5-8), *p* = 0.013) and the clinical outcome was worse (NIHSS 24 h after EST was higher in patients treated with NM (median, IQR; NM+: 14, 5–21 vs. NM-: 9, 3–18; *p* = 0.004), delta-mRS was higher in the NM + group (median, IQR; NM+: 3, 1–4 vs. NM-: 2, 1–2; *p* = 0.011)). Any ICH (NM+: 27/79, 34.2% vs. NM-: 356/1204, 29.6%; *p* = 0.386) and symptomatic ICH (NM+: 2/79, 2.5% vs. NM-: 21/1204, 1.7%; *p* = 0.609) was equally distributed between groups.

**Conclusion:**

Intra-arterial nimodipine during EST resolves iatrogenic vasospasms efficiently during EST without increasing intracranial hemorrhage rates. However, patients with vasospasms and NM treatment show higher infarct growth resulting in lower ASPECTS in follow-up imaging.

## Introduction

Intra-arterial administration of calcium channel blockers as nimodipine (NM) are widely used for the treatment of cerebral vasospasms. Nimodipine is a dihydropyridine agent that blocks voltage-gated calcium channels and has a dilatory effect on arterial smooth muscle. It is the only FDA-approved agent for vasospasms with a half-life of about nine hours [1]. Until now NM has been extensively studied in patients with subarachnoid hemorrhage and associated vasospasms. Also, NM has shown to resolve vasospasms resulting from endovascular stroke treatment (EST), where it is used as an off-label therapy [2]. Vasospasm as complication of intracranial mechanical thrombectomy is known as long as the therapy itself with the first case report published in 2009 [3]. It is a common iatrogenic complication during EST and can be located in the cervical access vessel as well as in the cerebral target vessel [4]. A review study by Balami et al. reported cervical or cerebral vasospasm during EST in 3 to 23% of cases [5, 6]. After the establishment of EST as a first-line therapy for acute ischemic stroke, the early randomized studies of 2015 did not thoroughly report on vasospasms during EST [7]. Since then, more studies have addressed the issues of procedure failure and interventional complications in EST [8]. As there are no systematic data available, the indication and dosage of intra-arterial NM in EST is currently at the discretion of the interventionalist. National or international standardized treatment protocols concerning this treatment option do not exist. Also, there is a lack of knowledge concerning the safety profile of NM for the treatment of vasospasms occurring during EST. Neurointerventionalists might be hesitant to apply a vasodilatative medication in stroke patients inducing increased cerebral blood flow in the downstream potentially infarcted territory and consecutively intracranial hemorrhage (ICH) in ischemic brain tissue [9]. Also, the effect of vasospasms despite the use of intra-arterial nimodipine on infarct development after EST is still unknown.

The aim of this study was to determine whether intra-arterial NM is an effective and safe treatment option for iatrogenic vasospasms during EST, in particular if it resolves vasospasms in angiographic imaging during intervention or also limits the infarct growth in follow-up imaging after EST. Moreover, we studied the impact of NM treatment on the patients’ clinical outcome.

## Methods

All consecutive patients with acute ischemic stroke in the anterior circulation and at least one intracranial thrombectomy attempt were selected from a prospective institutional review-board approved database of a tertiary stroke center in Germany treated between January 2015 and December 2021.

### EST and treatment protocol for vasospasms

The choice of material and primary thrombectomy approach (contact aspiration or stent retriever thrombectomy under continuous aspiration) were left to the interventionalist’s discretion. As an institutional standard approach, a triaxial system was used comprising a balloon-guided catheter (Merci 9 F 95 cm or Flowgate 8 F 95 cm, Stryker, Kalamazoo, USA), an intermediate catheter (e.g. Sofia 5 F/6F, Microvention, Aliso Viejo, California, USA) and a microcatheter/microwire system (most often Rebar18 and Traxcess14, Microvention, Aliso Viejo, California). The two stent retrievers being used most in this study are Solitaire X (Medtronic, Irvin, CA) and Trevo (Stryker, Kalamazoo, USA).

Vasospasms are graded differently across the literature. For this study, the grading of Kerz et al. was applied considering a decrease in vessel diameter of at least 70% as severe vasospasm prompting treatment [10].

**Fig. 1:**
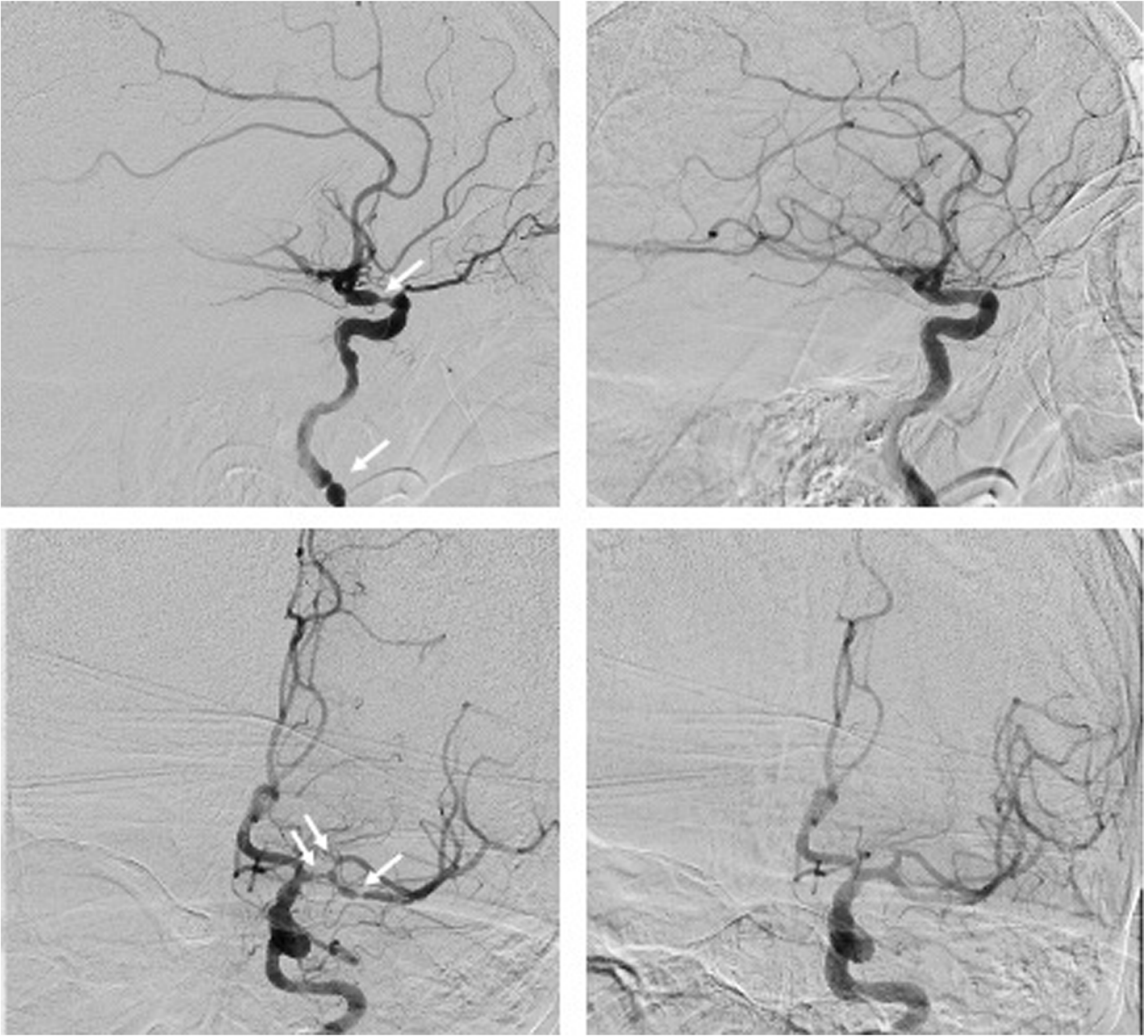
Left sided images show vasospasm in the internal carotid artery (top) and middle cerebral artery (bottom) after stent retriever thrombectomy before application of intra-arterial nimodipine. Right sided pictures show the same two patients 10 min after application of 1.5 mg nimodipine with a complete resolution of vasospasms

The treatment protocol for severe vasospasms after thrombectomy during EST in this institution stipulates intra-arterial NM infusion over an intermediate catheter or microcatheter placed in the appropriate parent vessel (M1-segment of middle cerebral artery or internal carotid artery) with a rate of 0.1 mg / min – see Fig. 1. The blood pressure is closely monitored during NM treatment to prevent temporary hypotension and the infusion rate is adjusted in case of any change in blood pressure.

### Study endpoints and patient selection

The primary endpoint of this study was cerebral infarction according to the Alberta Stroke Program Early CT Score (ASPECTS) in follow-up imaging 24 h (+/- 6 h) after EST [11].

Secondary endpoints of the study were short-term clinical outcome (NIHSS 24 h after EST and delta mRS, defined as the difference of mRS before stroke and at discharge) and intracranial hemorrhage (ICH) according to the Heidelberg bleeding classification (HBC) [12].

**Fig. 2:**
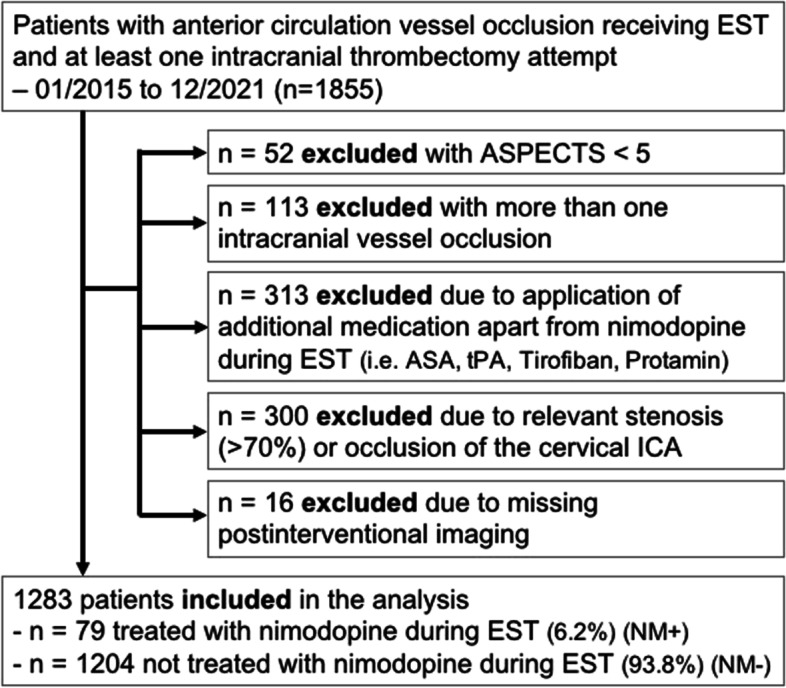
Patient selection for comparison of patients with vasospasms and intra-arterial nimodipine treatment (NM+) vs. no vasospasms or nimodipine during endovascular stroke treatment (NM-); ASA, acetylsalicylic acid; ASPECTS, Alberta Stroke Program early CT Score; EST, endovascular stroke treatment; ICA, internal carotid artery; tPA, tissue Plasminogen Activator

Exclusion criteria were (i) Alberta Stroke Program Early CT Score (ASPECTS) below 5 (ii) more than one intracranial target vessel occlusion or tandem occlusion with relevant stenosis or occlusion of the parent vessel (iii) application of intra-arterial or blood thinning intravenous medication other than NM during EST and (iv) missing follow-up imaging after EST – see Fig. 2. For the 16 patients excluded for missing postinterventional imaging, none of them received intra-arterial NM during EST, 7 of them died before imaging within the first 24 h after EST, 9 of them were transferred to the referring hospital before postinterventional imaging because their neurological symptoms resolved substantially.

### Study groups and statistical analysis

The two study groups were defined as iatrogenic vasospasms and intra-arterial NM infusion during EST (NM+) and no vasospasms and NM during EST (NM-). The study groups were compared in univariate analysis comprising relevant clinical and imaging data. Normal distribution was tested for each variable using the Shapiro-Wilk test. A Mann-Whitney U test or Chi-Square test was used as appropriate to compare the study groups in univariate analysis. For all statistical tests the significance level was set to *p* = 0.05. Medians are given with their interquartile range (IQR). All confidence intervals (CI) are quoted as 95%-CI. Statistical analyses were performed with SPSS Version 28 (IBM, Armonk, New York).

### Data acquisition

Source data were generated from a prospectively collected stroke database. Additionally, all data included in the present analysis were validated retrospectively to minimize incorrect or missing data (JJ, CW). The EST’s angiographic imaging was reviewed to assess the occurrence and potential resolution of cervical and cerebral vasospasm under the treatment with intra-arterial NM (JJ, CW). For assessing revascularization success and/or reperfusion after treating vasospasms, the modified Thrombolysis In Cerebral Infarction (mTICI) Score was applied [13]. Also, clinical data (e.g., medical records) were reviewed to assess the occurrence of neurological symptoms associated with ICH (JJ). The mRS and NIHSS are routinely assessed on the institution’s certified stroke unit or intensive care unit by trained neurologists.

## Results

In this study cohort of *n* = 1283 patients with acute ischemic stroke and EST, 79 patients (6.2%) developed vasospasms during EST and received intra-arterial NM. In all cases with NM + the medication was applied due to vasospasms:18/79 (22.8%) had cervical vasospasms (extracranial internal carotid artery), 69/79 (87.3%) had cerebral vasospasms (intracranial carotid artery or middle cerebral artery M1-4 segment or anterior cerebral artery) and 8/79 (10.1%) had combined cervical and cerebral vasospasms. On average, doses between 0.5 mg and 3 mg (median, IQR; NM dosage in mg: 1, 1–1.5) intra-arterial NM were applied. The targeted vasospasms resolved completely leaving no vessel stenosis or substantially improved with a persistent stenosis of not more than 10% in 66/79 patients (83.5%).

In this study NM + patients were younger (median, IQR, age in years; NM+: 66, 56–79 vs. NM-: 78, 68–84; *p* < 0.001), less likely to have cardiovascular risk factors such as arterial hypertension (NM+: *n* = 48, 61% vs. NM-: *n* = 933, 78%; *p* = 0.003) and less likely to take anticoagulation or antiplatelet medication before hospital admission for acute stroke treatment (NM+; 50 patients, 63% vs. NM-: 563, 47%) – see Table 1.

There were more patients with an unknown symptom onset in the NM + study group. However, the time windows for the acute treatment phase, including onset to imaging and imaging to groin puncture time did not differ between study groups – see also Table 1. Group differences in outcome can therefore not be related to differing time windows.

Stroke patients developing vasospasms and receiving intra-arterial NM during EST had a lower mRS before stroke (median, IQR, mRS; NM+: 0, 0–1 vs. NM-: 1, 0–2; *p* = < 0.001). While the study groups had a comparable ASPECTS before EST (median, IQR, ASPECTS; NM+: 8, 7–10 vs. NM-: 9, 8–10; *p* = 0.254), the ASPECTS in follow-up imaging was lower for patients with vasospasms treated with NM (median, IQR, ASPECTS follow-up; NM+: 6, 4.5–8 vs. NM-: 7, 6–9; *p* = 0.013) – see Table 1. The target vessel occlusion in NM- patients was more often a middle cerebral artery (MCA) occlusion in the M1-segment (NM+: *n* = 30, 38% vs. NM-: *n* = 583, 48%; *p* = 0.072), while in NM + patients MCA occlusions in the M2-segments were more frequent (NM+: *n* = 28, 37% vs. NM-: *n* = 370, 31%; *p* = 0.266). In average one additional thrombectomy maneuver with stent retrievers was performed, when patients developed vasospasms and were treated with intra-arterial NM (median, IQR, stent retriever maneuvers; NM+: 2, 1–3 vs. NM-: 1, 1–3; *p* < 0.001).

**Table 1:** Group comparison of patients treated with Nimodipine (NM+) or not (NM-). Bold values are statistically significant *p*-values (< 0.05) and their associated Odd’s Ratio; * Fisher exact test

	NM- (*n* = 1204)	NM+ (*n* = 79)	*p*-value
Age [years], median (IQR)	79 (69–84)	66 (55.6–78.5)	**< 0.001**
Male, n (%)	496 (41.2)	36 (45.6)	0.445
Coronary artery disease, n (%)	326 (27.0)	9 (12.9)	**0.003**
Known atrial fibrillation, n (%)	561 (46.4)	23 (29.1)	**0.003**
Arterial Hypertension, n (%)	933 (77.5)	48 (60.8)	**0.003**
Type 2 diabetes mellitus, n (%)	296 (24.6)	11 (13.9)	**0.038**
Hypercholesterolemia, n (%)	404 (33.6)	8 (10.1)	**< 0.001**
**Medication before stroke**			
None of the below mentioned, n (%)	563 (46.8)	50 (63.3)	**0.004**
Anticoagulation Vit. K Antagonist, n (%)	136 (11.3)	6 (7.6)	0.310
Direct Oral Anticoagulant, n (%)	187 (15.5)	8 (10.1)	0.195
Antiplatelet medication, n (%)	345 (28.7)	15 (19.0)	0.107
**Stroke related clinical and imaging aspects**			
Pre-stroke mRS, median (IQR)	1 (0–2)	0 (0–1)	**< 0.001**
Initial NIHSS score, median (IQR)	15 (9–20)	14 (9–19)	0.221
ASPECTS baseline, median (IQR)	9 (8–10)	8 (7–10)	0.254
ASPECTS follow-up, median (IQR)	7 (6–9)	6 (4.5-8)	**0.013**
Intravenous thrombolysis, n (%)	549 (45.6)	37 (46.8)	0.831
Unknown stroke onset, n (%)	452 (37.5)	38 (48.1)	**0.039**
Time from symptom onset to imaging, median [minutes], median (IQR)	206 (92–382)	219 (86–559)	0.558
Time from imaging to groin puncture, median [minutes], median (IQR)	40 (29–54)	41(31–55)	0.499
**Location of target vessel occlusion**			
Distal ICA, n (%)	44 (3.7)	4 (5.0)	0.523
Carotid T, n (%)	164 (13.6)	14 (17.7)	0.307
MCA M1 segment, n (%)	583 (48.4)	30 (38.0)	0.072
MCA M2 segment, n (%)	370 (30.7)	29 (36.7)	0.266
MCA M3/ M4 segment, n (%)	21 (1.7)	2 (2.9)	0.609
ACA, n (%)	11 (0.9)	0	0.394
**Procedural aspects**			
Treatment in conscious sedation, n (%)	1042 (86.5)	70 (88.6)	0.932
Number of thrombectomy attempts in total, median (IQR)	2 (1–3)	3 (2–5)	**< 0.001**
Number of stent retriever maneuvers, median (IQR)	1 (1–3)	2 (1–3)	**< 0.001**
Number of aspiration maneuvers, median (IQR)	0 (0–1)	0 (0–1)	0.099
**mTICI after treatment**			
0–1, n (%)	38 (3.2)	5 (7.1)	0.129
2a, n (%)	68 (5.6)	8 (10.1)	0.184
2b, n (%)	332 (27.6)	21 (26.6)	0.412
2c-3, n (%) (Reperfusion)	766 (63.6)	42 (53.2)	0.062
**Intracranial hemorrhage in follow-up imaging**			
Any intracranial hemorrhage, n (%)	356 (29.6)	27 (34.2)	0.386
Symptomatic hemorrhage, n (%)	21 (1.7)	2 (2.5)	0.609
**Heidelberg Bleeding Classification (HBC)**			
Scattered small petechiae, no mass effect HBC 1a, n %	81 (6.5)	6 (7.6)	0.766
Confluent petechiae, no mass effect HBC 1b, n %	78 (6.5)	7 (8.9)	0.410
Hematoma within infarcted tissue, occupying < 30%, no substantive mass effect HBC 1c, n %	75 (6.2)	5 (6.3)	0.973
Intracerebral hemorrhage within and beyond infarcted brain tissue HBC 2, n %	33 (2.7)	1 (1.3)	0.429
Parenchymal hematoma remote from infarcted brain tissue HBC 3a, n %	5 (0.4)	0	0.566
Intraventricular hemorrhage; HBC 3b, n %	29 (2.4)	0	0.163
Subarachnoid hemorrhage; HBC 3c, n %	141 (11.7)	13 (16.5)	0.209
Subdural hemorrhage; HBC 3d, n %	3 (0.2)	0	0.657
**Other complications**			
Perforation, n %	33 (2.7)	3 (3.8)	0.582
Dissection, n %	9 (0.7)	0	0.441
Embolism in new territory, n %	9 (0.7)	2 (2.5)	0.062*
Embolism in same territory, n %	5 (0.4)	2 (2.5)	0.304
**Clinical Outcome**			
mRS at discharge, median IQR	4 (2–5)	4 (2–5)	0.192
Delta mRS, median IQR	2 (1–2)	3 (1–4)	**0.011**
NIHSS 24 h, median (IQR)	9 (3–18)	14 (5–21)	**0.004**
NIHSS at discharge, median (IQR)	6 (2–17)	10 (2.5–17.5)	0.144

Follow-up imaging after EST showed no increased incidence of any intracranial hemorrhage (NM+: *n* = 27, 34% vs. NM-: *n* = 356, 30%; *p* = 0,386) or symptomatic ICH (NM+: 2 patients, 3% vs. NM-: 21, 2%; *p* = 0.609), but a lower ASPECTS in patients treated with NM (see above) and a higher NIHSS 24 h after EST (median, IQR, NIHSS 24 h; NM+: 14, 5–21 vs. NM-: 9, 3–18; *p* = 0.004) was observed. There were no other complications like dissection or perforation associated with NM infusion. The clinical outcome measured by the mRS at discharge was not different between the study groups (median, IQR, mRS discharge; NM+: 4, 2–5 vs. NM-: 4, 2–5; *p* = 0.192). However, the difference of mRS before stroke and mRS at discharge (delta mRS) was higher in the NM + group (median, IQR, delta mRS; NM+: 3, 1–4 vs. NM-: 2, 1–2; *p* < 0.011).

## Discussion

EST has become the treatment of choice in anterior circulation ischemic stroke patients with large vessel occlusions with a relatively low risk of periprocedural complications including iatrogenic vasospasm. In this study cohort, EST patients developed vasospasms and received intra-arterial nimodipine (NM) infusion in 6% of all cases, which is in line with the rate of vasospasms during EST reported in previous publications [3–7]. Angiographically, NM resolved the targeted vasospasm in the majority of cases (84%) in this cohort. The prophylactic treatment with NM to prevent vasospasms during EST is routinely performed by many interventionalists individually. However, there is a lack of evidence concerning this treatment approach, which is not performed at the study facility.

Nimodipine was not associated with a higher rate of symptomatic or asymptomatic intracerebral hemorrhage. Hemorrhagic transformation and intracerebral hemorrhage after ischemic stroke are related to blood extravasates across a disrupted blood brain barrier triggered by risk factors like hypertension, hyperglycemia, and age [14]. Our study results imply that i.a. NM does not contribute to a higher risk of ICH, e.g. by increase in brain perfusion.

We found, that patients with vasospasms and NM therapy are younger and overall healthier before stroke onset. Comparably, younger patients are also prone to develop vasospasms after subarachnoid hemorrhage (SAH). In the setting of SAH, vasospasms are triggered by biochemical reactions caused, inter alia, by the disintegrated hemoglobin released from blood in the subarachnoid space [15]. Torbey et al. showed that younger patients with SAH develop vasospasms at a higher mean flow velocity in transcranial Doppler compared to older patients. Therefore, younger patients are more vulnerable for developing larger infarctions after SAH [16–19]. In patients receiving EST due to ischemic stroke, vasospasm in the target vessel is likely to be a reaction to mechanical stress caused by the use of thrombectomy devices. Thus, the etiology of vasospasms differs to SAH patients. The age-dependent decrease of vessel wall elasticity is discussed as cause for age-dependency of vasospasms in SAH patients and might also explain, that EST patients developing vasospasms are younger.

Our results are in line with Söderqvist et al., who also reported intracranial vasospasms more frequently after stent retriever maneuvers compared to contact aspiration, supporting the recommendation of McTaggart et al. to avoid further stent retriever maneuvers after the initial occurrence of iatrogenic vasospasms [20, 21].

Furthermore, vasospasms tend to be more frequent when treating more distal target vessel occlusions in this study. With medium vessel occlusions (MeVOs) being the designated new frontier in EST, the need for appropriate management of iatrogenic vasospasms seems pivotal. This holds especially true since more distal target vessel occlusions can most often not be reached by intermediate catheters for contact aspiration but only with stent retrievers. If newly developed smaller aspiration catheters are also less prone to cause vasospasms compared to small stent retrievers remains to be seen. Although, vasospasms are detected and can be treated effectively immediately during EST, the infarct growth in follow-up imaging after EST was higher resulting in a lower ASPECTS compared to patients without vasospasms. A possible explanation for this finding could be the re-occlusion of spastic vessels after the EST. These patients might profit from a closer diagnostic monitoring (CT-perfusion imaging or transcranial Doppler sonography) after the acute treatment phase.

We acknowledge the limitations of this study related to the single-center and retrospective design. Also, the complex interaction between the occurrence of vasospasm and its effective and lasting treatment are unclear. We do not know if the resolution of vasospasm after NM treatment during EST is sustained in the days after acute ischemic stroke care. Therefore, more studies are warranted to define, if intra-arterial NM improves merely the angiographic result by resolving iatrogenic vasospasm or if spastic vessels tend to re-occlude after EST resulting in more extensive ischemic lesions. Also, differing local treatment protocols regarding vasospasms might influence the safety profile of nimodipine or other medication to treat vasospasms. As all of the patients in our study cohort received NM for the treatment of vasospasms, the lower ASPECTS in follow-up imaging is driven by the occurrence of iatrogenic vasospasms itself and not by the treatment with NM. Also, the higher increase of mRS after EST in these patients underlines the role of vasospasms as relevant and underestimated complication of endovascular stroke treatment.

## Conclusion

Intra-arterial nimodipine application in iatrogenic vasospasms during endovascular stroke treatment is angiographically speaking an effective treatment without increasing the rate of intracranial hemorrhage in this study. Although nimodipine resolved vasospasms in the majority of patients in this study, the ASPECTS in follow-up imaging was lower and the short-term outcome worse compared to patients without vasospasms. This calls for further investigation on the influence of vasospasm during EST on infarct progression and clinical outcome.

## Data Availability

The datasets generated and/or analyzed during the current study are not publicly available due to local regulations and restrictions but are available from the corresponding author on reasonable request.
